# Histopathological biomarkers in squamous cell carcinoma of the vulva: the prognostic relevance of tumor-infiltrating lymphocytes (TILs)—a retrospective study of 157 cases

**DOI:** 10.1007/s12672-025-02381-x

**Published:** 2025-04-19

**Authors:** Gilbert Georg Klamminger, Meletios P. Nigdelis, Yaman Degirmenci, Bashar Haj Hamoud, Erich Franz Solomayer, Laura Schnöder, Bernd Holleczek, Marcus Schmidt, Annette Hasenburg, Mathias Wagner

**Affiliations:** 1 Department of General and Special Pathology, Saarland University (USAAR), Saarland University Medical Center (UKS), 66424 Homburg, Germany; 2 Department of Obstetrics and Gynecology, University Medical Center of the Johannes Gutenberg University Mainz, Langenbeckstraße 1, 55131 Mainz, Germany; 3 Department of Gynecology, Obstetrics and Reproductive Medicine, Saarland University Medical Center (UKS), 66424 Homburg, Germany; 4 Saarland University Medical Center for Tumor Diseases (UTS), Homburg, Germany; 5 Saarland Cancer Registry, 66117 Saarbrücken, Germany

**Keywords:** Vulvar cancer, Tumor-infiltrating lymphocytes, TILs, Histological biomarker, Immunooncological biomarker

## Abstract

**Background:**

The prognostic relevance of tumor-infiltrating lymphocytes (TILs) has so far been recognized in several solid tumors like in breast, endometrial and ovarian cancer—nonetheless, the immune contexture of squamous cell carcinomas of the vulva, analyzed by means of stromal (s) and intratumoral (i) TILs, remains yet to be elucidated.

**Material and methods:**

In this study, we examined the immunooncological biomarkers sTILs and iTILs in 157 vulvectomy specimens with histologically diagnosed vulvar squamous cell carcinoma (VSCC) according to the standardized methodology proposed by the *International Immunooncology Biomarkers Working Group* in 2017. In a next step, we evaluated the association of infiltrating lymphocytes to traditional histopathological parameters such as infiltration depth and HPV related tumorigenesis. After determining optimal cut-off values using Receiver Operating Characteristic (ROC) curve analysis, we assessed the prognostic relevance of sTILs and iTILs with regard to overall survival, local recurrence, and metastasis using the Log-rank (Mantel–Cox) test and Fisher's exact test.

**Results:**

We propose optimal cut-off values of 5% for iTILs and 20% for sTILs analysis, which identify patients with a distinct superior survival rate (sTILs: *p* = 0.0137; iTILs: *p* = 0.0226). Furthermore, a low number of iTILs was associated with a higher risk of local recurrence in our study collective (*p* = 0.0432).

**Conclusion:**

The fast and cost-effective determination of the histological biomarkers iTILs and sTILs yields prognostic relevance in vulvar cancer. A potential integration within the routine diagnostic workflow could be globally feasible, even in resource-poor settings.

**Supplementary Information:**

The online version contains supplementary material available at 10.1007/s12672-025-02381-x.

## Introduction

From an oncological perspective the host immune response—which has long been recognized in solid tumors by pathologists when microscopically assessing stromal responses and thus the tumor microenvironment next to the infiltrating tumor areas—is increasingly relevant for two reasons. First, the phenotypical aspects of the so-called immune contexture (the organized distribution of immune reactions in proximity to neoplastic cell clusters, histologically recognizable as tumor-infiltrating lymphocytes (TILs)) may serve as a prognostic and predictive factor [1, 2]—the beneficial role of histological TIL assessment as immunooncological biomarker in various solid neoplasms as well as their integration within the routine pathological report has already been proposed [3, 4]. Secondly, immunotherapeutic approaches and the individual patient’s respondence may directly be linked to antigenic properties of the tumor; assessment of the immune contexture may therefore not only serve useful to evaluate treatment options but also potentially to overcome drug resistances to cancer immunotherapies [5].

As one outstanding histological biomarker, investigation of TILs is a relatively easy assessable and cost-effective tool to evaluate the host immune response directly on hematoxylin and eosin (H&E) stainings during routine histopathological diagnostics. Nevertheless, differing methods of assessment modalities as well as diverging scoring systems among different entities are hampering a broader implementation into the routine clinical workflow and urged the *International Immunooncology Biomarkers Working Group* in 2017 to publish a practical review, proposing a general and standardized method of TIL assessment in solid neoplasms [6, 7]. Albeit previous studies evaluated the prognostic impact of TILs in gynecological malignancies just as breast, endometrial and ovarian cancer—interestingly, researchers hereby even determined associations with distinct molecular tumor properties (such as POLE-mutations, MSS/I (microsatellite stable/instable) status, or BRCA1-mutation) [8–15]—a broader and systematic research evaluating the prognostic relevance of stromal (s) and intratumoral (i) TILs within squamous cell carcinomas of the vulva (VSCC) employing the systematic methodology as proposed by the above-mentioned *International Immunooncology Biomarkers Working Group* has not yet been conducted. We postulate a prognostic relevance of infiltrating immune cell infiltrates also within vulvar cancer and hence examined the prognostic value of sTILs and iTILs in a cohort of 157 patients with primary VSCC with regard to metastasis, local recurrence, and overall survival.

## Materials and methods

Patients with primary VSCC who underwent surgical treatment and consecutive histopathological diagnosis at the University Hospital of Saarland (Germany) within the years 2007–2023 were screened for study inclusion according to beforehand defined inclusion and exclusion criteria; see also Supp. Table 1. Relevant clinical and morphological information such as age or histopathological tumor characteristics (tumor stage, infiltration depth, perineural invasion, vascular space invasion, groin lymph node metastasis, p16-status/HPV-status wherever feasible) were retrieved by chart review of the electronic databases of the pathology unit (Department of General and Special Pathology, University Hospital of Saarland, Germany). In order to allow comparability between previously used versions of relevant classification systems of the past, that have been updated over the years, all patient information required for this study such as TNM stage or classification of VSCC tumor type (HPV-associated/HPV-independent/not otherwise specified NOS) was restated according to the actual 8th edition of the TNM classification of malignant tumors (2018) and the new *2020 WHO Classification of Female Genital Tumors* [16]. Study approval for this retrospective study was retrieved by the Ethics Committee of Saarland, Germany (study identification number 249/23, approved on 7 March 2024). Study reporting was based on the STrengthening the Reporting of OBservational studies in Epidemiology (STROBE) guideline [17]. Relevant oncological follow-up data (overall survival, local recurrences, development of metastasis) were made available by the statewide-active ‘Saarland Cancer Registry’ and the ‘Medical Center for Tumor Diseases’ at Saarland University.

The semiquantitative evaluation of sTILs and iTILs was performed in best accordance with latest recommendations previously proposed by the *International Immunooncology Biomarkers Working Group* in a practical review, which ultimately represent an adaptation of the guidelines for TIL evaluation in breast cancer and previous studies of Denkert et al. [18, 19]; the general reproducibility of these standardized methodology has been recently reported [20]. Diagnostic H&E stained tissue samples of included cases were assessed on a multi-head light microscope; to minimize interobserver variability, consensus was reached for each case by consensus-seeking discussion of interpreting authors.

**Fig. 1:**
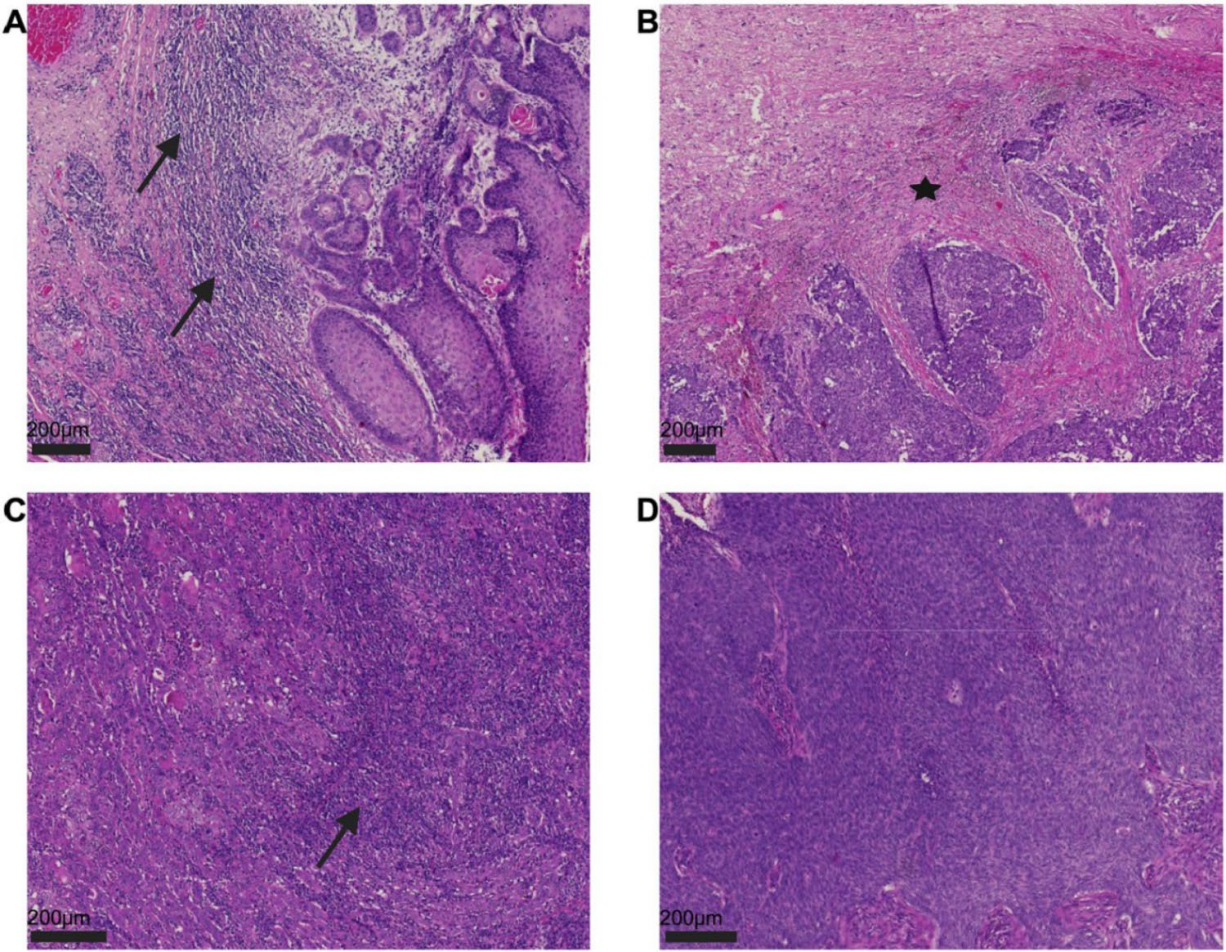
**A** Next to infiltrating tumor areas within the stromal compartment there is a dense lymphocytic infiltrate (≥ 20% per peritumoral stromal rim; sTILs marked by arrow signs). **B** Depiction of a case with < 20% sTILs within the area of the invasive stromal margin (indicated by a black star). **C** Within the intratumoral compartment, a dense lymphocytic infiltrate can be determined (iTILs ≥ 5% per tumoral area). **D** Central tumor areas of a vulvar carcinoma without relevant intratumoral inflammatory cell infiltrate. **A**–**D** All hematoxylin and eosin staining

For each case, TILs—defined as all mononuclear cells such as plasma cells or lymphocytes while all neutrophils, eosinophils, and basophils as polymorphonuclear leukocytes were not considered—were separately assessed within the ‘stromal compartment’ of the so-called *invasive margin*, a 1-mm rim between neoplastic infiltrating cells and peritumoral host tissue (sTILs) as well as the ‘tumor cell compartment’ (iTILs) on at least five fields of view using a × 20 objective. TILs exceeding the range of the tumor border as well as TILs within areas of prior biopsy or necrotic tissue were not considered. TILs were reported as % per either stromal or tumoral area; Fig. 1 and Supp. Figure 1 present different variations of TILs. During analysis, the Supplementary Material provided by Hendry et al. served, as intended, as reference material for scoring of TILs [6].

Statistical analysis was performed employing the GraphPad software (Boston, MA 02110, US). After descriptive statistics and an initial normality check (Shapiro–Wilk test) were carried out, a search for statistically relevant correlations between TILs (as defined, TILs are initially considered a continuous variable) and traditional clinicopathological parameters was conducted using Spearman Rho analysis.

Defining a *sTILs low group* as sTILs < 20% was achieved by using the overall median of 20% within our sTILs study cohort as cut-off value; correspondingly the *sTILs high group* was defined as sTILs ≥ 20%. Since for iTILs the range within our study cohort was solely between min. 0% to max. 10% we determined the optimal cut off using a Receiver Operating Characteristic (ROC) curve analysis and consecutive evaluation of the Youden’s Index (YI) for each threshold. To assess the relevance in terms of overall survival, the Log-rank (Mantel–Cox) test was used; differences between unpaired groups were determined using Fisher's exact test. For all tests, statistical significance was defined using the threshold of α < 0.05.

## Results

Our entire study cohort comprised 157 patients with a median age of 66 years (interquartile range: 53–79), meeting a priori defined inclusion criteria; a subset of nine patients were excluded. In total 30 (19.1%) patients were diagnosed with pT1a tumor, 108 (68.8%) patients with a pT1b tumor and 19 (12.1%) patients with a pT2 tumor. While 124 (79.0%) cases did not show inguinal lymph node metastasis, in 33 (21.0%) of cases groin lymph node metastasis could be determined. 25 (15.9%) tumors showed HPV-association, 54 (34.4%) were classified as HPV-independent, and 78 (49.7%) were reported as squamous cell carcinoma of the vulva NOS (not otherwise specified). An overview of clinicopathological characteristics is displayed in Table 1.

**Table 1:** Clinical characteristics of our study cohort

Clinical characteristics/variables	*N* = 157
Age, years	66 (median; 95% IQR: 53–79)
Histological type: HPV-associated	25 (15.9%)
Histological type: HPV-independent	54 (34.4%)
Histological type: not otherwise specified	78 (49.7%)
T1a	30 (19.1%)
T1b	108 (68.8%)
T2	19 (12.1%)
N0	124 (79.0%)
Positive groin lymph node affection (Nmic/N1a to N2c)	33 (21.0%)
L0	132 (84.1%)
L1	25 (15.9%)
V0	146 (93.0%)
V1	11 (7.0%)
Pn0	144 (91.1%)
Pn1	13 (8.3%)
Infiltration depth, cm	0.7134 (mean), 0.8120 (std. deviation)
Median follow up time, months	34 (median; IQR: 14–72.5)
sTILs < 20%	76 (48.4%)
sTILs ≥ 20%	81 (51.6%)
iTILs < 2%	115 (73.2%)
iTILs ≥ 2%	42 (26.8%)
iTILs < 5%	142 (90.4%)
iTILs ≥ 5%	15 (9.6%)

*CI* confidence interval; *IQR* interquartile range

While sTILs range from 1% to 80%, iTILs range from 0% to 10%; both did not show a normal data distribution within initial normality check (sTILs and iTILs: *p* < 0.0001). While an increasing percentage of stromal lymphatic response is negatively correlated with age (Spearman correlation: *r* = −0.1632; *p* = 0.0412), tumor stage (Spearman correlation: *r* = −0.2092; *p* = 0.0085), and infiltration depth (Spearman correlation: *r* = −0.2643; *p* = 0.0008) we did not determine any correlation with vascular/lymphatic/perineural invasion. Interestingly, iTILs only showed a correlation with age (Spearman correlation: *r* = −0.2420; *p* = 0.0023). Neither of our parameters put to test did show a significant association to tumorigenesis, namely HPV association (Spearman correlation, sTILs: *r* = −0.0024; *p* = 0.9832; iTILs: *r* = 0.0368; *p* = 0.7474; see Supp. Table 2). Aiming to evaluate the optimal cut-off value for iTILs analysis, we determined the highest YI for iTILs < 2% (YI:0.14) and iTILs < 5% (YI:0.12). In order to verify our proposed cut-off value for sTILs analysis we furthermore re-run the ROC curve analysis also for this parameter and indeed demonstrated an optimal sensitivity/specificity trade-off of at around 20% (highest YI for: sTILs 17.5% = 0.18 and sTILs 22.5% = 0.12).

**Fig. 2:**
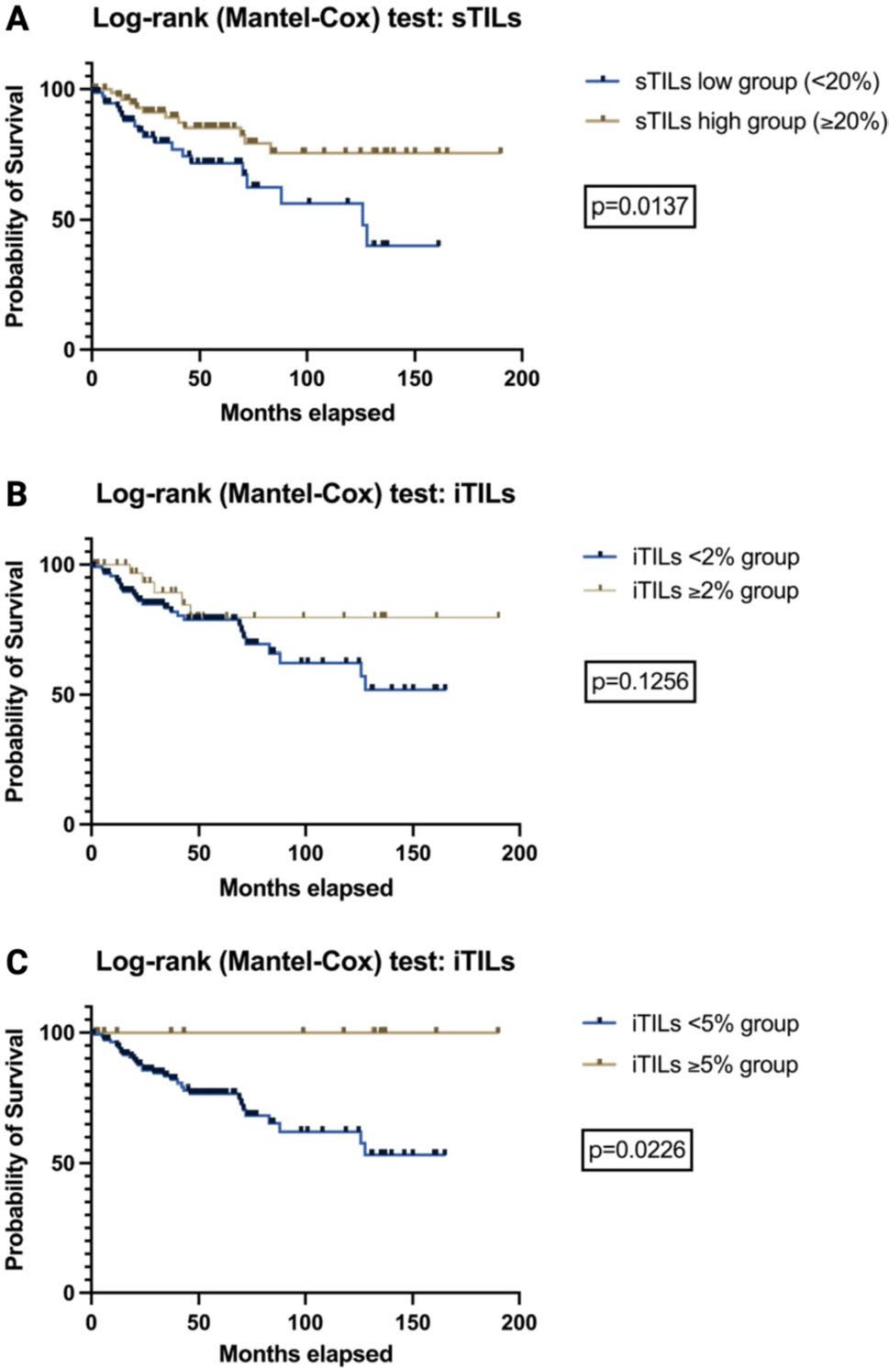
Survival curves of VSCC patients in accordance to different group splitting: *sTILs high group vs. sTILs low group* (**A**), *iTILs* < *2% vs. iTILs* ≥ *2%* (**B**), and *iTILs* < *5% vs. iTILs* ≥ *5%* (**C**)

Analyzing the impact of sTILs on overall survival using univariate analysis, we determined a significant benefit for patients within the *sTILs high group* (Log-rank test, *p* = 0.0137; *x*^2^ = 6.073; Fig. 2A). While the proposed cut-off value for iTILs group separation of < 2% did not show any significant results (Log-rank test, *p* = 0.1256; Fig. 2B), the cut-off value of < 5% showed a distinct benefit for the iTILs high group (Log-rank test, *p* = 0.0226; *x*^2^ = 5.199; Fig. 2C). For additional details on follow-up periods for each group defined see also Supp. Table 3.

While risk of metastasis did not show any differences neither when comparing groups in accordance to sTILs (*sTILs high group* vs. *sTILs low group;* Fisher's exact test; *p* = 0.7822) nor to iTILs (< 5% group vs. ≥ 5% group, Fisher's exact test; *p* = 0.3644), risk of local recurrence was significantly reduced in patients with iTILs ≥ 5% (< 5% group vs. ≥ 5% group, Fisher's exact test; *p* = 0.0432). Contrasting, high infiltration of sTILs did not show lower rates of recurrences (*sTILs high group* vs. *sTILs low group;* Fisher's exact test; *p* > 0.9999); see Tables 2 and 3.

**Table 2:** Group comparison of *sTILs high group vs. sTILs low group* in accordance to local recurrence as well as development of metastasis

Contingency table analysis	Fisher's exact test: *p* value (two-tailed)
Development of recurrence (*sTILs high group vs. sTILs low group*)	*p* > 0,9999
Development of metastasis (*sTILs high group vs. sTILs low group*)	*p* = 0.7822

**Table 3:** Group comparison of *iTILs* < *5% group vs.* ≥ *5% group* in accordance to local recurrence as well as development of metastasis

Contingency table analysis	Fisher's exact test: *p* value (two-tailed)
Development of recurrence (*iTILs* < *5% group vs.* ≥ *5% group*)	*p* = 0.0432
Development of metastasis (*iTILs* < *5% group vs.* ≥ *5% group*)	*p* = 0.3644

## Discussion

In this study we assessed sTILs and iTILs in VSCC on regular H&E sections according to the methodology of the *International Immunooncology Biomarkers Working Group*, in order to evaluate their prognostic relevance as histological biomarker. Hence, we demonstrate superior survival rates in patients with sTILs ≥ 20% as well as with iTILs ≥ 5%. While the prognostic impact of iTILs < 5% is also represented in higher rates of local recurrences but not metastasis in this particular subgroup, sTILs are neither associated with risk of recurrence nor metastasis.

From a histomorphological point of view, reports recognizing differing inflammatory cell infiltration patterns (sometimes also referred to as *lymphoplasmocytic infiltration*) in individual vulvar tumors trace back already decades ago [21–23]. In the absence of a standardized definition and method, some research groups examined the clinico-pathological role of inflammatory cell infiltrates surrounding tumor tissue; hereby, most previous studies ultimately failed to show a prognostic benefit of the inflammatory response studied e.g., in terms of inguinal lymph node metastasis [24–26]. Solely few approaches aimed at a more advanced evaluation of distinct immune reactions in VC, using an immunohistochemical approach which sub-classifies different subsets of infiltrating lymphocytes. Although they partly differ in their study design (evaluation of tissue-micro arrays vs. whole slide analysis or different immunohistochemical markers put to test), the vast majority of them did not report an influence of certain (sub-)populations of lymphocytes with regard to prognosis [27–30]: De Jong et al. did not determine an association of CD8 + T-lymphocytes and Foxp3 + T-lymphocytes with regard to survival when examining specimens of 286 patients with vulvar cancer [27]. The team of Sznurkowski et al. evaluated the presence of CD4 + and CD8 + TILs in 76 samples of VSCC and indeed noted distinct clusters not only intratumoral but also within stromal areas; however, albeit only intratumoral TILs were subsequently evaluated and showed a positive correlation (Spearman analysis: *r* = 0.282, *p* = 0.014) between CD4 + and CD8 + cells, none of the analyzed subgroups provided prognostic information regarding patient survival [28]. In another study, this research group examined intratumoral regulatory T cells (Foxp3 +) in primary VSCC as well as lymph node metastasis; their intensity was not associated with overall survival rates [30]. Contrasting, Kortekaas et al. demonstrated superior survival rates in vulvar cancer patients with intense t-cell infiltration (e.g., helper T cells defined by CD3 + positivity and CD8 and Foxp3 negativity) regardless of the HPV status when measuring lymphocytes per cells/mm^2^ in intraepithelial but also stromal compartments within VSCCs [31, 32]. That said, even previous approaches already noted and partly aimed at evaluation of inflammatory cells within differing intratumoral and stromal areas—a ratio not only reflected within the actual recommendation of the *International Immunooncology Biomarkers Working Group* but also based on the lack of a current deeper knowledge on the biological interrelations between sTILs and iTILs. Albeit an H&E slide-based analysis does neither allow for further immunohistochemical classification of lymphocytic differentiation nor mRNA based functional assessment of the transcriptome, a comparable prognostic value is presumed [6, 15, 19].

To our knowledge this is the first time TILs analysis was performed in vulvar cancer according to the standardized method proposed by the *International Immunooncology Biomarkers Working Group—*our study demonstrated a distinct prognostic value assessing TILs in VSCC. With a total number of 157 VSCC included, we furthermore evaluated a sufficiently sized cohort of a rare gynecological tumor entity [33, 34]. All immunooncological parameters analyzed in this study are globally accessible even in resource-poor settings, using only H&E sections. In comparison to more time-consuming and cost-intensive techniques such as mRNA expression profiling, assessment of TILs is a cost-effective and fast performable analysis, that could be easily integrated within the routine diagnostic workflow and the pathological report. From a scientific perspective, the above-mentioned standardized method of TILs evaluation can be easily transferred also to other rare solid tumor types of the female lower genital tract such as squamous neoplasms of the vagina.

In this project we did not determine the functionals aspects of the histological immune contexture studied; therefore (patho-)physiological antitumor responses or immunological tumor-host interactions may be more sufficiently characterized using more advanced approaches such as DNA and RNA analysis [35]. Additionally, immunohistochemical approaches that subclassify the immune cell infiltrate may derive insights into the distinct value of for example dendritic cells too [1]. Last but not least the relevance of TILs in accordance to cancer immunotherapy as well as its impact on response to neoadjuvant chemotherapy using solely biopsy material for biomarker evaluation has not be evaluated within the scope of this study [36]. Future research approaches could also evaluate the prognostic relevance of TILs within metastatic tumor deposits, with a special attention paid to upcoming difficulties when evaluating immune cell infiltrates e.g., within lymph node metastasis [37]. Within future studies the standardized methodology proposed by the *International Immunooncology Biomarkers Working Group* should explicitly be evaluated for any potential intra- and interobserver variability when evaluating TILs in VSCC; however, recent studies suggest a general high scoring agreement [38]. Within this study, we aimed at maximized consensus of TIL quantification evaluating the histological immune response on a multi-head light microscope in a shared-diagnostic approach; the data we present are well suitable e.g., for future meta-analysis.

With the standardized evaluation of iTILs and sTILs we propose a fast and low-cost analysis tool for the immune contexture in VSCC. As a histological immunooncological biomarker associated not only with overall survival rates but also local recurrence, mention of its appearance within the microscopical description of the pathological reports would not only enable further research due to a greater accessibility of this biomarker but also a future interinstitutional comparability.

## Supplementary Information

Additional file1 (DOCX 929 KB)

## Data Availability

Please contact the corresponding author for individual solutions.
