# Supplementary material for: Histopathological biomarkers in squamous cell carcinoma of the vulva: the prognostic relevance of tumor-infiltrating lymphocytes (TILs)—a retrospective study of 157 cases
Source: Discov Oncol. 2025 Apr 19;16:572. doi: 10.1007/s12672-025-02381-x (PMC12009255; doi:10.1007/s12672-025-02381-x)
Supplement: Supplementary file 1 — Additional file1 (DOCX 929 KB) [file 12672_2025_2381_MOESM1_ESM.docx]

**SUPPLEMENTARY MATERIAL**

**Title:** Histopathological Biomarkers in Squamous Cell Carcinoma of the Vulva: The Prognostic Relevance of Tumor-Infiltrating Lymphocytes (TILs) - A Retrospective Study of 157 Cases

| **Inclusion criteria** | **Exclusion criteria** |
| --- | --- |
| Squamous cell carcinoma of the vulva | precursor lesions / intraepithelial lesions |
| oncological follow up data available (occurrence of metastasis, recurrence; overall survival) | recurrent vulvar carcinomas |
| Vulvectomy/wide excision specimen available for pathological diagnosis | solely biopsy material available |
| - | vulvar tumors other than squamous cell carcinomas |

**Supp. Table 1**. Defined criteria for study inclusion/exclusion.

| **Spearman correlation:**  **p16/HPV status** in association to - | **r** | 95% confidence interval | **p value (two-tailed)** |
| --- | --- | --- | --- |
| sTILs | -0.0024 | -0.2297 to 0.2251 | 0.9832 |
| iTILs | 0.0368 | -0.1922 to 0.2620 | 0.7474 |

**Supp. Table 2.** Spearman correlation did not show any significant association of sTILs and iTILs with p16/HPV status.

| **defined groups (number of patients included)** | **median survival time, months** |
| --- | --- |
| sTILs<20% (n=76) | 26.5 (median; 95% CI: 15-42) |
| sTILs ≥20% (n=81) | 48 (median; 95% CI: 31-61) |
| iTILs<2% (n=115) | 34 (median; 95% CI: 23-54) |
| iTILs ≥2% (n=42) | 35 (median; 95% CI: 21-49) |
| iTILs<5% (n=142) | 33 (median; 95% CI: 24-46) |
| iTILs ≥5% (n=15) | 99 (median; 95% CI: 12-136) |

**Supp. Table 3.** Depiction of follow-up periods according to each sTILs/iTILs group.


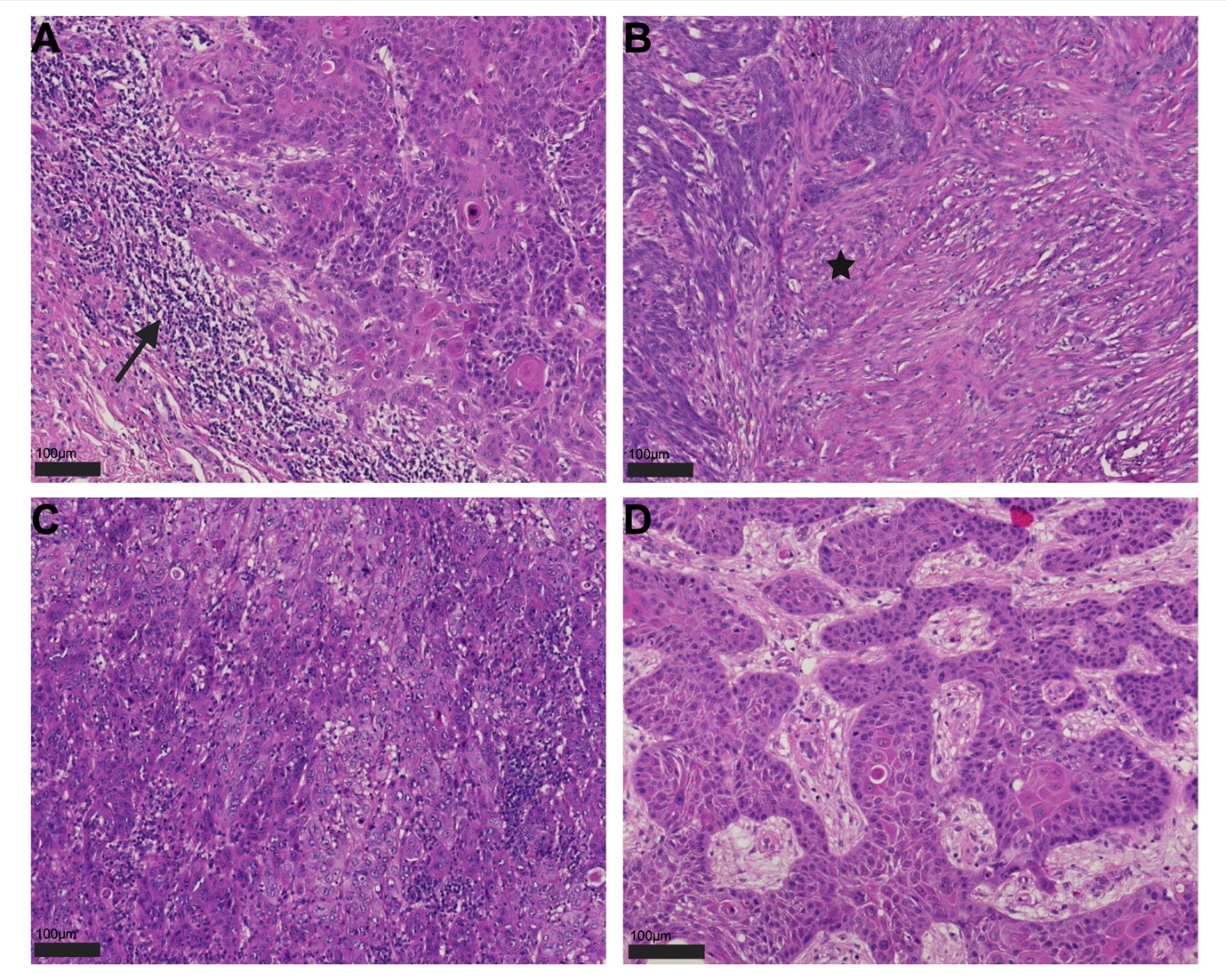


**Supp. Figure 1.** Visualization of the investigated biomarkers (TILs), here displayed in larger magnification than in Figure 1. **A**: sTILs high (≥20% per peritumoral stromal rim; sTILs marked by arrow signs). **B**: sTILs low (<20% sTILs within the area of the invasive stromal margin, indicated by a black star). **C**: iTILs appr. 5% per tumoral area. **D**: iTILs <5% per tumoral area. **A-D:** All hematoxylin and eosin staining.
